# Know your foe: synanthropic spiders are deterred by semiochemicals of European fire ants

**DOI:** 10.1098/rsos.210279

**Published:** 2021-05-19

**Authors:** Andreas Fischer, Yerin Lee, T'ea Dong, Gerhard Gries

**Affiliations:** Department of Biological Sciences, Simon Fraser University, Vancouver, British Columbia, Canada

**Keywords:** ant cue, spider deterrent, integrated pest management, *Myrmica rubra*

## Abstract

Many ants prey on spiders, suggesting that web-building spiders may avoid micro-locations near ant colonies or frequented by foraging ants. Here we tested the hypothesis that ant-derived semiochemicals deter synanthropic spiders. To generate stimuli, we exposed filter paper for 12 h to workers of European fire ants, *Myrmica rubra,* black garden ants, *Lasius niger,* or western carpenter ants, *Camponotus modoc*, and then offered select urban spiders in three-chamber olfactometer bioassays a choice between ant-exposed filter paper and unexposed control filter paper. Semiochemical deposits of *M. rubra*, but not of *L. niger* or *C. modoc*, had a significant deterrent effect on subadults of the false black widow, *Steatoda grossa,* the black widow, *Latrodectus hesperus*, and the hobo spider, *Eratigena agrestis,* as well as a moderate (but statistically not significant) deterrent effect on the cross spider, *Araneus diadematus*. The deterrent effect caused by semiochemical deposits of *M. rubra* may be attributable to the aggressive nature and efficient foraging of *M. rubra* in its invaded North American range, exerting selection pressure on community members to recognize *M. rubra* semiochemicals and to avoid micro-locations occupied by *M. rubra*.

## 1. Introduction

Widespread arachnophobia [1,2] is fuelled, in part, by fear of the few neurotoxic spiders [3,4]. This fear has inspired the development of tactics to physically and chemically discourage synanthropic spiders from settling in and around human dwellings [5]. Proposed physical tactics include sealing holes and cracks in building walls, removing webs, reducing moisture and changing exterior lighting that attracts insect prey for spiders [3]. Chemical tactics such as insecticide applications [5] are largely ineffective because spiders can avoid insecticides by abandoning their web and rebuilding one elsewhere [3,6]. Natural repellents of spiders, such as chestnuts and lemon oil, are widely advertised in anecdotal accounts but only a few have been experimentally tested [7,8], and none effectively repelled all species of spiders tested [9]. Moreover, there is no immediate ecological reason why these materials are repellent to spiders.

By contrast, there is every reason for spiders to avoid natural predators such as ants that prey on both web-building and cursorial spiders [10–12]. At the population level, there is a negative correlation between the density of ant populations and the total biomass of spiders [13,14]. Cobweb spiders, *Phylloneta impressa,* tend to disperse in response to chemical cues derived from black garden ants, *Lasius niger,* and the formicine ant *Formica clara* [15]. Sensing chemical cues of potentially predatory ants is particularly adaptive for subadult web-building spiders which seek suitable micro-locations for settling and building their webs [16]. As web building is a significant time and energy investment [17–19], subadult spiders are thought to explore, and ultimately select, primarily those microhabitats that have no or few threats to survival, such as the presence of predatory ants. Flat rock spiders, *Morebilus plagusius*, e.g. avoid ant-scented rocks when selecting retreat sites [20].

Here we tested the hypothesis that ant-derived semiochemicals deter spiders. As model organisms for our study, we selected three synanthropic ant species [European fire ants, *Myrmica rubra*; black garden ants, *Lasius niger;* western carpenter ants, *Camponotus modoc* (all Formicidae)] and four synanthropic web-building spider species [false black widow, *Steatoda grossa;* western black widow, *Latrodectus hesperus* (both Theridiidae); cross spider, *Araneus diadematus* (Araneidae); hobo spider, *Eratigena agrestis* (Agelenidae)], all of which are commonly found in and around human dwellings in North America [21].

## 2. Material and methods

### 2.1. Ants

**Figure 1.**
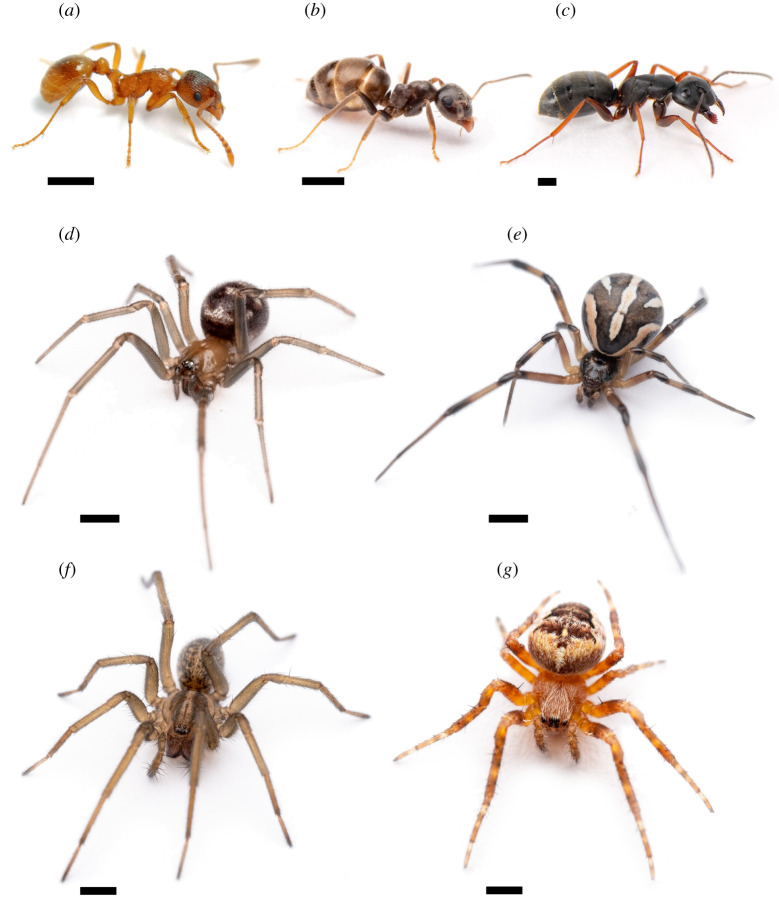
Photographs of worker ants of *Myrmica rubra* (*a*), *Lasius niger* (*b*) and *Camponotus modoc* (*c*) that were used to prepare test stimuli, and of subadult female spiders of *Steatoda grossa* (*d*), *Latrodectus hesperus* (*e*), *Eratigena agrestis* (*f*) and *Araneus diadematus* (*g*) that were tested in laboratory experiments. Bar length, 1 mm.

*Myrmica rubra* workers (figure 1) were collected from nests at Inter River Park (49°19'10.9″ N 123°01'43.7″ W) in North Vancouver, British Columbia (BC), Canada, whereas workers of *L. niger* and *C. modoc* (figure 1) were collected from nests located on the Burnaby campus of Simon Fraser University (SFU, 49°16'33″ N 122°54'55″ W), BC. All ants were kept in jars (1–4 l) filled with soil from collection sites and were provisioned with tubes of sugar water retained with a cotton ball. To standardize the presentation of test stimuli according to weight equivalent of ants, 75 workers of each species were weighed in groups of five using a microbalance (TR-204, Denver Instrument Comp., Arvada, CO 80004, USA). Body weights (mean ± s.e.) of individual workers of *M. rubra, L. niger* and *C. modoc* amounted to 3.51 ± 5.56, 3.02 ± 4.44 and 43.7 ± 52.7 mg, respectively.

### 2.2. Spiders tested

All specimens of *S. grossa* (figure 1) were F_1_ subadult offspring of mated females captured on SFU's Burnaby campus [22], whereas specimens of *L. hesperus* and *E. agrestis* (figure 1) were F_1_ subadult offspring of mated females collected on Centennial Beach Boundary Bay Regional Park, Delta, BC (49°01'10.9″ N 123°02'32.1″ W). Spiderlings were housed singly in a Petri dish (100 × 20 mm) containing a moist cotton wick and—based on body size—were provisioned with *Drosophila* vinegar flies or *Phormia regina* blow flies once a week.

All *A. diadematus* were subadults, collected on the day of bioassays on SFU's Burnaby campus. Following bioassays, they were released into a designated non-collection zone on campus.

### 2.3. General experiments design

The effects of ant-derived deposits on aversion responses by spiders were tested in still-air, dual-choice olfactometers [8,23] kept at room temperature and a 12 L : 12 D photoperiod. Olfactometers (see fig. 1 in [8] for a photographic illustration) consisted of three circular Pyrex glass chambers (3.5 × 10 cm inner diameter (ID)) with removable glass lids linearly interconnected by glass tubes (each 2.5 × 1 cm ID). The bottoms of lateral chambers were lined with circular filter paper (Whatman, Maidstone, England). Treatment and control stimuli were assigned to lateral chambers such that the treatment stimulus was equally often presented in the left and right lateral chamber of an olfactometer to minimize any potential effect of side bias. To prepare a treatment stimulus, ants were placed in one lateral chamber and prevented from leaving by a wet cotton ball inserted in the glass tube interconnecting the lateral and central chamber. The wet cotton ball not only blocked the chamber exit, but also provided a source of moisture for the ants. To ensure symmetry of the experimental design, a wet cotton ball was also inserted in the glass tube interconnecting the central chamber and the second lateral chamber. As the quantity of semiochemicals deposited by ants was probably correlated with their body size or weight, equal weight equivalents of ants were used to standardize the preparation of treatment stimuli; hence, 37 *M. rubra,* 43 *L. niger* and 3 *C. modoc* were confined in the treatment chamber. After 12 h of (overnight) confinement, the ants and the cotton balls were removed. Then, a bioassay spider was introduced into the central chamber and kept in darkness for 24 h, following which its final position was scored under red light. Spiders positioned in lateral chambers were classed as responders to treatment or control stimuli, whereas those in the central chamber were recorded as non-responders. Spiders located in an interconnecting glass tube were scored as non-responders if they were closer to the central chamber than to the respective lateral chamber. All spiders were tested only once, and olfactometers were washed in detergent water (Sparkleen, Fischerbrand, Toronto, Canada) and oven-dried between replicates.

### 2.4. Specific experiments

Experiment 1 (table 1) was designed to reveal potential side bias associated with olfactometers. It tested the response of *S. grossa* to two control stimuli (untreated filter paper) which were presented in the lateral chambers of the olfactometer.

**Table 1.** List of test stimuli consisting of filter paper with chemical deposits from the ants *Myrmica rubra*, *Lasius niger* or *Camponotus modoc*, and of control stimuli invariably consisting of filter paper without chemical deposits by any ants, tested for behavioral responses of the synanthropic spiders *Steatoda grossa*, *Latrodectus hesperus*, *Eratigena agrestis* and *Araneus diadematus* in binary choice olfactometer experiments.

Exp. no	test stimulus^a^	control stimulus	spider species bioassayed	*n*^b^
bioassays with *S. grossa* to test for potential side bias of olfactometers				
1	no deposits	no deposits	*S. grossa*	24 (3)
effect of ant species-specific chemical deposits on behavioural responses of *S. grossa*				
2	37 *M. rubra*	no deposits	*S. grossa*	24 (3)
3	43 *L. niger*	no deposits	*S. grossa*	24 (1)
4	3 *C. modoc*	no deposits	*S. grossa*	24 (2)
effect of *M. rubra* chemical deposits on behavioural responses of four synanthropic spiders				
5	37 *M. rubra*	no deposits	*S. grossa*	30 (9)
6	37 *M. rubra*	no deposits	*L. hesperus*	30 (11)
7	37 *M. rubra*	no deposits	*E. agrestis*	30 (8)
8	37 *M. rubra*	no deposits	*A. diadematus*	30 (11)
effect of *M. rubra* chemical deposit amounts on behavioural responses of *S. grossa*				
9	37 *M. rubra*	no deposits	*S. grossa*	30 (7)
10	111 *M. rubra*	no deposits	*S. grossa*	30 (10)

^a^Equal weight equivalents of ants (37 *M. rubra*, 43 *L. niger* and 3 *C. modoc*) were used to standardize the preparation of test stimuli (chemicals deposited by ants on filter paper during 12 h).

^b^*n* = number of replicates run (number of spiders not responding in bioassays).

As there was no side bias in experiment 1 (see Results), experiments 2–4 (table 1) then tested whether semiochemicals deposited by *M. rubra* (Exp. 2), *L. niger* (Exp. 3) or *C. modoc* (Exp. 4), have a deterrent effect on *S. grossa*.

As only semiochemical deposits of *M. rubra*, but not of *L. niger* or *C. modoc,* deterred *S. grossa* (see Results), follow-up experiments 5–8 (table 1) focused on *M. rubra* semiochemicals, and tested whether they deter only *S. grossa* (Exp. 5), or also deter *L. hesperus* (Exp. 6), *E. agrestis* (Exp. 7) and *A. diadematus* (Exp. 8).

With evidence that *M. rubra* semiochemicals deter at least three spider heterogeners (see Results), experiments 9 and 10 (table 1) then tested dose-dependent effects of deterrent semiochemicals by offering *S. grossa* a choice between filter paper left untreated (control) or soiled with semiochemicals from either 37 *M. rubra* workers (Exp. 9; the same dose as in Exps. 3, 5–8) or 111 *M. rubra* workers (Exp. 10; a threefold higher dose).

### 2.5. Statistical analysis

R [24] was used to perform one-sided binominal tests to analyse data for the hypothesized repellent effect of ants on spiders in two choice experiments 1–10 [25]. Subsequently, the *p*-values of experiments were adjusted using the Benjamini–Hochberg method to account for multiple comparisons [26].

## 3. Results

**Figure 2.**
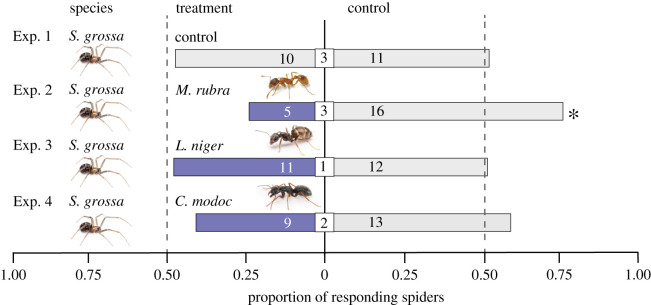
Responses of subadult *Steatoda grossa* that were given a choice in three-chamber olfactometers [23] between two test stimuli, both being untreated filter paper (Exp. 1), or one, being untreated filter paper, and the other being filter paper previously exposed to worker ants of *Myrmica rubra* (37; Exp. 2), *Lasius niger* (43; Exp. 3), or *Camponotus modoc* (3; Exp. 4). Shown within bars and square inserts are the number of spiders responding to treatment or control stimuli, and not responding to stimuli, respectively. For each experiment, an asterisk (*) denotes a statistically significant treatment effect (one-sided binomial tests; *p* < 0.05).

**Figure 3.**
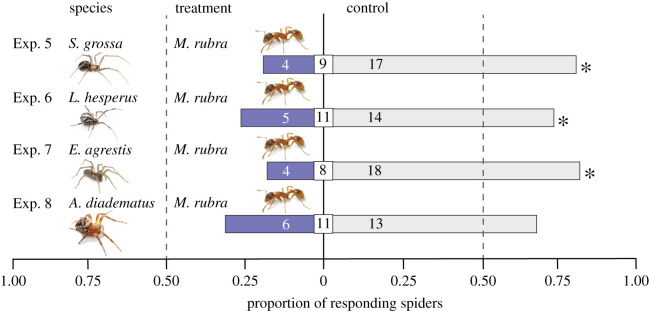
Responses of subadult *Steatoda grossa* (Exp. 5), subadult *Latrodectus hesperus* (Exp. 6), subadult *Eratigena agrestis* (Exp. 7), and subadult *Araneus diadematus* (Exp. 8) that were given a choice in three-chamber olfactometers [23] between two test stimuli, one being untreated filter paper and the other being filter paper previously exposed to 37 worker ants of *Myrmica rubra.* Shown within bars and square inserts are the number of spiders responding to treatment or control stimuli, and not responding to stimuli, respectively. For each experiment, an asterisk (*) denotes a statistically significant treatment effect (one-sided binomial tests; *p* < 0.05).

**Figure 4.**
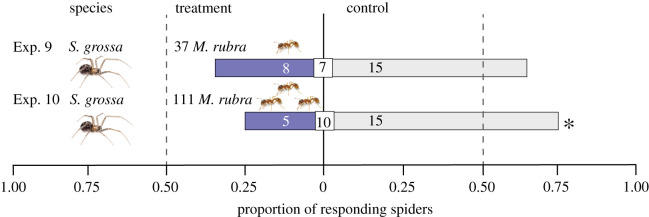
Responses of subadult *Steatoda grossa* that were given a choice in three-chamber olfactometers [23] between two test stimuli, one being untreated filter paper and the other being filter paper previously exposed to 37 or 111 worker ants of *Myrmica rubra* (Exps. 9 and 10, respectively). Shown within bars and square inserts are the number of spiders responding to treatment or control stimuli, and not responding to stimuli, respectively. For each experiment, an asterisk (*) denotes a statistically significant treatment effect (one-sided binomial tests; *p* < 0.05).

When subadult *S. grossa* were offered a choice between two lateral olfactometer chambers, each containing a control stimulus (untreated filter paper), they chose the right and left chamber 11 and 10 times, respectively, revealing no evidence for a side bias (*p* *=* 0.50; Exp. 1, figure 2). Semiochemicals deposited by *M. rubra* had a significant deterrent effect on *S. grossa* (Exp. 2: spiders in treatment chamber (5) versus spiders in control chamber (16), *p* *=* 0.004; figure 2). By contrast, semiochemicals deposited by *L. niger* (Exp. 3) or *C. modoc* (Exp. 4) failed to deter *S. grossa* (Exp. 3: 11 versus 12; *p* *=* 0.50; Exp. 4: 9 versus 13, *p* *=* 0.393; figure 2). In parallel experiments 5–8, semiochemicals deposited by *M. rubra* had a significant deterrent effect on *S. grossa* (Exp. 5: 4 versus 17; *p* *=* 0.007, figure 3), *L. hesperus* (Exp. 6: 5 versus 14; *p* *=* 0.042, figure 3) and *E. agrestis* (Exp. 7: 4 versus 18; *p* *=* 0.007, figure 3), but not on *A. diadematus* (Exp. 8: 6 versus 13; *p* *=* 0.084, figure 3). There was a dose-dependent effect of the amount of semiochemicals deposited by *M. rubra* on behavioural responses of *S. grossa.* The amount of semiochemicals deposited by 111 *M. rubra* had a deterrent effect on *S. grossa* (Exp. 10: 5 versus 15, *p* *=* 0.041, figure 4) but the aversion effect caused by deposits of only 37 *M. rubra* was not statistically significant in this particular experiment (Exp. 9: 8 versus 15; *p* *=* 0.105, figure 4).

## 4. Discussion

Our data support the conclusion that semiochemical deposits of *M. rubra* worker ants have a significant deterrent effect on three spider species (*S. grossa*, *L. hesperus, E. agrestis*) and that they express a moderate deterrent effect on a fourth spider species tested in our study, the cross spider *A. diadematus*. Conversely, semiochemical deposits of *L. niger* and *C. modoc* worker ants failed to induce a discernible behaviour-modifying effect on the spiders tested.

Our findings that semiochemical deposits of *M. rubra* worker ants, but not of *L. niger* or *C. modoc* worker ants, prompted aversion responses by *S. grossa* have multiple potential explanations, such as the specifics of the experimental design, contrasting life-history traits of ants, and niche overlap, or not, between ants and spiders.

As part of the experimental design to prepare ant semiochemical deposits as test stimuli for spiders, we selected diverse taxonomic species of ants that greatly varied in body size and weight. Assuming that larger ants deposit greater amounts of semiochemicals, we standardized the amount of deposits between experiments by testing equal weight equivalents of ants, using 37, 43 and 3 worker ants of *M. rubra, L. niger* and *C. modoc*, respectively, to generate a test stimulus. However, contrary to our assumption, the body weight of ants and the amount of semiochemical deposits may not be positively correlated, and equal numbers, rather than equal weights, of *M. rubra, L. niger* and C*. modoc* worker ants may have been required to generate standardized test stimuli. Alternatively, the semiochemicals deposited by *M. rubra* may have significantly greater potency as spider deterrents than those of *L. niger* and *C. modoc*. Worker ants of *M. rubra* are omnivorous scavengers and prey on many invertebrates [27]. In their invaded North American range, populations of *M. rubra* occur in extremely high densities and appear more aggressive than their counterparts in Europe. These characteristics, coupled with efficient foraging and aggressive nest defence, have enabled *M. rubra* to outcompete native ants and lower the arthropod biodiversity in invaded communities [28]. It is conceivable then, that over evolutionary time arthropod community members, including spiders which may fall prey to *M. rubra*, have learned to respond to semiochemical cues of *M. rubra* and to settle in (micro) habitats void of *M. rubra*. If so, this would provide ecological rationale for our data showing that semiochemical deposits of *M. rubra* have deterrent effects on *S. grossa*, *L. hesperus* and *E. agrestis*.

Insufficient niche overlap between *M. rubra* and *A. diadematus,* and thus a lack of opportunity to learn each other's semiochemical signals or cues, may explain why semiochemical deposits of *M. rubra* had only a weak (and statistically not significant) deterrent effect on *A. diadematus*. As orb-weavers, *A. diadematus* females build their webs above ground [29], physically well separated from the subterranean colonies of *M. rubra*. Females of *S. grossa*, *L. hesperus* and *E. agrestis*, in contrast, build their three-dimensional cobwebs near ground level [29] with greater likelihood of frequent encounters with foraging *M. rubra* workers.

The identity of the deterrent semiochemical(s) deposited by *M. rubra* workers remains unknown. Communication signals such as trail or alarm pheromones [30–34] are least likely to be the deterrent(s) because the sets of 37 *M. rubra* workers used to generate test stimuli in the confines of olfactometers had no immediately obvious incentive to release pheromone and coordinate activities. Yet, signalling in ants is complex and we are just beginning to grasp that complexity. While the functional role of most exocrine glands in *M. rubra* [35] is still unknown, any gland may have released the semiochemical(s) that prompted the deterrent effect on spiders. Alternatively, the semiochemicals are not released from glands but originate from the ants' body surface.

Irrespectively, the rather remarkable deterrence of *M. rubra* semiochemical deposits against *S. grossa*, *L. hesperus* and *E. agrestis* warrant the identification of these deterrents through proven-effective techniques in arthropod chemical ecology [36]. Once identified, the origin of these deterrents could be traced to a specific exocrine gland and/or the body surface of ants. Moreover, synthetic replica of these deterrents could be developed, together with concurrently known spider deterrents [9], for earth-friendly manipulation of synanthropic spiders.

## Data accessibility

All data are presented in the manuscript and in the electronic supplementary material [37].

## References

[1] Marks I. 1987 Fears, phobias and rituals: panic, anxiety and their disorders, 1st edn. Oxford, UK: Oxford University Press.

[2] Davey GCL. 2008 The ‘disgusting’ spider: The role of disease and illness in the perpetuation of fear of spiders. Soc. Anim. **2**, 17–25. (10.1163/156853094X00045)

[3] Vetter RS. 2011 Spiders. In Handbook of pest control (ed. A Mallis), pp. 1082–1117. Richfield, OH: The Mallis Handbook Company.

[4] Hauke TJ, Herzig V. 2017 Dangerous arachnids—fake news or reality? Toxicon **138**, 173–183. (10.1016/j.toxicon.2017.08.024) 28866287

[5] Ramires EN, Navarro-Silva MA, Marques FDA. 2011 Chemical control of spiders and scorpions in urban areas. In Pesticides in the modern world – pests control and pesticides exposure and toxicity assessment (ed. M Stoycheva), pp. 553–600. Rijeka, Croatia: InTech.

[6] Pekár S. 2012 Spiders (Araneae) in the pesticide world: an ecotoxicological review. Pest Manag. Sci. **68**, 1438–1446. (10.1002/ps.3397) 22945871

[7] Fischer A, Ayasse M, Andrade MCB. 2018 Natural compounds as spider repellents: fact or myth? J. Econ. Entomol. **111**, 314–318. (10.1093/jee/tox339) 29309619

[8] Fischer A, MacLennan S, Gries R, Gries G. 2021 Herbivore-induced plant volatiles do not affect settling decisions by synanthropic spiders. Chemoecology. (10.1007/s00049-021-00340-w)

[9] Fischer A. 2019 Chemical communication in spiders – a methodological review. J. Arachnol. **47**, 1–27. (10.1636/0161-8202-47.1.1)

[10] Gillespie RG, Reimer N. 1993 The effect of alien predatory ants (Hymenoptera: Formicidae) on Hawaiian endemic spiders (Araneae: Tetragnathidae). Pacific Sci. **47**, 21–33.

[11] Henschel JR. 1998 Predation on social and solitary individuals of the spider *Stegodyphus dumicola* (Araneae, Eresidae). J. Arachnol. **26**, 61–69. (10.2307/3705451)

[12] Jackson RR, Nelson XJ, Pollard SD, Edwards GB, Barrion AT. 2004 Predation by ants on jumping spiders (Araneae: Salticidae) in the Philippines. New Zeal. J. Zool. **31**, 45–56. (10.1080/03014223.2004.9518358)

[13] Sanders D, Platner C. 2007 Intraguild interactions between spiders and ants and top-down control in a grassland food web. Oecologia **150**, 611–624. (10.1007/s00442-006-0538-5) 17091284

[14] Halaj J, Ross DW, Moldenke AR. 1997 Negative effects of ant foraging on spiders in Douglas-fir canopies. Oecologia **109**, 313–322. (10.1007/s004420050089) 28307185

[15] Mestre L, Bucher R, Entling MH. 2014 Trait-mediated effects between predators: ant chemical cues induce spider dispersal. J. Zool. **293**, 119–125. (10.1111/jzo.12127)

[16] Foelix R. 2015 Biologie der Spinnen, 3rd edn. Frankfurt, Germany: Chimaira.

[17] Tanaka K. 1989 Energetic cost of web construction and its effect on web relocation in the web-building spider *Agelena limbata*. Oecologia **81**, 459–464. (10.1007/BF00378952) 28312637

[18] Janetos AC. 1982 Foraging tactics of two guilds of web-spinning spiders. Behav. Ecol. Sociobiol. **10**, 19–27. (10.1007/BF00296392)

[19] Fischer A, Hung E, Gries G. 2019 Female false black widow spiders, *Steatoda grossa*, recognize webs based on physical and chemical cues. Entomol. Exp. Appl. **167**, 803–810. (10.1111/eea.12825)

[20] Penfold S, Dayananda B, Webb JK. 2016 Chemical cues influence retreat-site selection by flat rock spiders. Behaviour **154**, 149–161. (10.1163/1568539X-00003415)

[21] Bradley RA. 2012 Common spiders of North America. Berkeley, CA: University of California Press.

[22] Fischer A, Lee Y, Stewart J, Gries G. 2018 Dodging sexual conflict? – Sub-adult females of a web-building spider stay cryptic to mate-seeking adult males. Ethology **124**, 838–843. (10.1111/eth.12807)

[23] Takács S, Gries G. 2001 Communication ecology of webbing clothes moth: attractiveness and characterization of male-produced sonic aggregation signal(s). Can. Entomol. **133**, 725–727. (10.1046/j.1439-0418.2003.00724.x)

[24] R Core Team. 2020 R: a language and environment for statistical computing. Vienna, Austria: R Foundation for Statistical Computing.

[25] Ruxton GD, Neuhäuser M. 2010 When should we use one-tailed hypothesis testing? Methods Ecol. Evol. **1**, 114–117. (10.1111/j.2041-210x.2010.00014.x)

[26] Chen SY, Feng Z, Yi X. 2017 A general introduction to adjustment for multiple comparisons. J. Thorac. Dis. **9**, 1725–1729. (10.21037/jtd.2017.05.34) 28740688 PMC5506159

[27] Reznikova ZI, Panteleeva SN. 2001 Interaction of the ant *Myrmica rubra* L. as a predator with springtails (Collembola) as a mass prey. Dokl. Biol. Sci. **380**, 475–477. (10.1023/A:1012383607521) 12918409

[28] Naumann K, Higgins RJ. 2015 The European fire ant (Hymenoptera: Formicidae) as an invasive species: Impact on local ant species and other epigaeic arthropods. Can. Entomol. **147**, 592–601. (10.4039/tce.2014.69)

[29] Bellmann H. 2010 Der Kosmos-Spinnenführer, 1st edn. Stuttgart, Germany: Kosmos.

[30] Cammaerts MC, Inwood MR, Morgan ED, Parry K, Tyler RC. 1978 Comparative study of the pheromones emitted by workers of the ants *Myrmica rubra* and *Myrmica scabrinodis*. J. Insect Physiol. **24**, 207–214. (10.1016/0022-1910(78)90036-7)

[31] Cammaerts MC, Evershed RP, Morgan ED. 1981 Comparative study of the mandibular gland secretion of four species of *Myrmica* ants. J. Insect Physiol. **27**, 225–231. (10.1016/0022-1910(81)90055-X)

[32] Cammaerts-Tricot MC. 1973 Pheromones agregeant les ouvrieres de *Myrmica rubra*. J. Insect Physiol. **19**, 1299–1315. (10.1016/0022-1910(73)90213-8)

[33] Evershed RP, Morgan ED, Cammaerts MC. 1982 3-Ethyl-2,5-dimethylpyrazine, the trail pheromone from the venom gland of eight species of *Myrmica* ants. Insect Biochem. **12**, 383–391. (10.1016/0020-1790(82)90035-X)

[34] Morgan ED, Wadhams LJ. 1972 Chemical constituents of Dufour's gland in the ant *Myrmica rubra*. J. Insect Physiol. **18**, 1125–1135. (10.1016/0022-1910(72)90147-3)

[35] Morgan ED. 2008 Chemical sorcery for sociality: exocrine secretions of ants (Hymenoptera: Formicidae). Myrmecol. News **11**, 79–90.

[36] Millar JG, Haynes KF. 1998 Methods in chemical ecology. Norwell, MA: Kluwer Academic Publishers.

[37] Fischer A, Lee Y, Dong T, Gries G. 2021 Know your foe – synanthropic spiders are deterred by semiochemicals of European fire ants. Figshare. 10.1098/rsos.210279 PMC8131949 34017604

